# The impact of precompetition state on athletic performance among track and field athletes using machine learning

**DOI:** 10.3389/fphys.2025.1429510

**Published:** 2025-02-07

**Authors:** Yuting Zhang, Pengyu Fu, Qi Yu, Qingmei Niu, Dongfeng Nie, Xiangya Dou, Xiaoqin Zhang

**Affiliations:** ^1^ College of Public Policy and Management, Northwestern Polytechnical University, Xi’an, Shaanxi, China; ^2^ Department of Physical Education, Northwestern Polytechnical University, Xi’an, Shaanxi, China; ^3^ College of Life Science, Northwestern Polytechnical University, Xi’an, Shaanxi, China; ^4^ Department of Physical Education, The Affiliated School of Shanxi Agricultural University, Tai’yuan, Shaanxi, China

**Keywords:** track and field athletes, pre-competition status, competition performance, machine learning, training monitoring

## Abstract

**Objective:**

This study aims to compare the differences in the precompetition status (nutritional, physiological, biochemical, psychological, and sleep statuses) among college track and field athletes with different competition performances and to screen for key indicators of differences affecting athletic performance.

**Methods:**

Multiple indicators, traditional methods, and machine learning methods are used to detect the exercise load, fatigue index, and precompetition state of athletes with different sports performances.

**Results:**

(1) Two weeks before the competition, the fat mass in the left upper limb in the BP group was significantly higher than that in the BnP group (*P* < 0.05). The absolute values of blood basophils and triglycerides (TGs) in the BnP group were significantly higher than those in the BP group (*P* < 0.05). The positive detection rate of urinary leukocytes in the BnP group was higher than that in the BP group, and the positive detection rate of urinary occult blood and vitamin C in the BP group was higher than that in the BnP group. (2) One week before the competition, the blood lactate dehydrogenase (LDH) in the BP group was significantly higher than that in the BnP group (*P* < 0.05). The detection rate of positive urinary occult blood in the BnP group was higher than that in the BP group (*P* < 0.05). (3) No significant differences were found in the daily dietary intake, energy consumption values, physical activity, sleep efficiency, real-time heart rate, real-time respiratory rate, and real-time heart rate variability between the intensive and reduced periods. (4) The Rosenberg Self-Esteem Scale score of the BnP group was significantly higher than that of the BP group (*P* < 0.05).

**Conclusion:**

Precompetition absolute basophil, LDH, TG, white blood cells, creatine kinase, fat mass in the left upper limb, erythrocyte pressure (HCT), and individual failure anxiety can be used as training monitoring indicators that focus on tracking athlete status before the race.

## 1 Introduction

Monitoring athlete status during training provides insight into health, fatigue, and recovery, which is important in assessing training adaptation and athletic performance (Halson, 2014). The rapid development of big data, cloud computing, the Internet of Things, and other technologies exposes all walks of life, including the sports industry, to the influence of digital technology (Glebova and Desbordes, 2021). Multi-indicator and multidimensional sports training monitoring have also gradually become a hotspot for athlete training monitoring. Wearable devices based on artificial intelligence algorithms and athlete evaluation systems are rapidly growing in the sports industry (Mateus, 2023). Wearable devices, such as accelerometers, are used to assess athletes’ physical activity and sleep, among other things (Ding, 2023). Real-time heart rate monitoring sensors and EEG signal analysis based on machine learning algorithms are also widely used in the athlete community (Ding, 2023; Jiang and He, 2023; Tan and Ren, 2023). Moreover, machine learning algorithms have been used as a data processing method for predicting athletes’ injuries, health status, psychological status, and athletic performance. However, most of the existing studies have focused on elite athletes. They also lack scientific training monitoring tools for college athletes. Moreover, few studies were conducted on groups of collegiate athletes. When machine learning algorithms are used for research, they are rarely used for training monitoring and determining the weighting of factors that affect athletes’ athletic performance (Miah et al., 2023; Li, 2023; McGuigan, 2017).

For the monitoring of college track races (sprints), the book *Monitoring Training and Performance in Athletes* (Loucks et al., 2013) was used in this study to select the relevant indicators for judging external load, internal load, and fatigue determination. Given the wide application of science and technology in the field of sports, training monitors can accurately monitor the external loads of athletes during training in real time. This study uses accelerometers and other equipment to obtain the external load data of athletes’ energy consumption and activity intensity. Monitoring only the external load of the athlete cannot accurately describe the physiological load and psychological load produced by the athlete in training and other indicators of the internal load. In this study, the real-time heart rate sensor and the psychological questionnaires are used to monitor the real-time heart rate of the athlete and the athlete’s pregame psychological state, respectively. Moreover, hormones and other biochemical indicators, such as cortisol, testosterone, and other indicators, are measured to monitor the athlete’s internal load and fatigue state.

On the basis of the current status and shortcomings of the previous research, this study monitors the above indicators of athletes 2 weeks before the competition (training period) and 1 week before the competition (reduction period). It also uses multinomial logistic regression, random forest algorithm in machine learning, and principal component analysis to assess the importance of the indicators. Established through neural networks (NNs), a prediction model of athletic performance is also discussed in this paper. Thus, machine learning research in the field of sports science is enriched. The weights of the indicators are evaluated to reveal the relationship between the precompetition state and performance, assess the degree of influence of the precompetition state on the performance, and optimize athletes’ precompetition state adjustment strategies. This study provides new methods and perspectives for the study of the relationship between pregame state and game performance through the application of machine learning algorithms. It also helps promote the development and innovation of related theories, enriches and improves the theoretical system of sports training, and promotes the scientific and precise development of sports training.

## 2 Materials and methods

### 2.1 Research objects and groups

Fifteen high-level track and field athletes from Northwestern Polytechnical University, including 10 men and 15 women, were selected as subjects for this study. The athletes were divided into groups to compare their performance in the Shaanxi Provincial University Athletics Championships with their performance in the National University Athletics Championships. Those who improved in individual events were classified as the performance improvement group (BP group; *n* = 8), whereas those who did not improve were classified as the performance decline group (BnP group, *n* = 7). The BP group included four women, whereas the BnP group included one woman. The subjects had an average age of 21.20 ± 1.74 years, an average training period of 6.8 ± 2.188 years, an average height of 175.56 ± 6.09 cm, and an average weight of 64.24 ± 7.58 kg. The inclusion criteria are as follows: having experience in regular sprint training and participation in the 2023 Shaanxi Provincial University Athletics Championships and the 2023 National University Athletics Championships. The exclusion criteria are as follows: recent injuries; unsuitability for preparation for competition; having taken antibiotics in the past 6 months; or having suffered from gastrointestinal issues, such as diarrhea or constipation. The subjects signed informed consent forms after understanding the content, potential risks, and benefits of the study. This study was approved by the Ethics Committee for Medical and Experimental Animals at Northwestern Polytechnical University (Ethics Approval No. 202302040).

### 2.2 Body composition testing

Body composition was measured 1 and 2 weeks before the race at the same time once every week. The subjects were asked to remain fasted early in the morning and to test at rest (no strenuous exercise and sufficient sleep [≥8 h] 24 h before testing).

### 2.3 Meal records

Athletes’ meals (including food types and estimated weights of three meals and additional meals) were recorded 1 week before the competition and 2 weeks before the competition using the 24 h retrospective method. The nutritional composition table of food was discussed, and the relative values of energy of the three major nutrients in each athlete’s daily dietary intake were calculated based on the energy coefficients of the three major energy-supplying nutrients (4 kcal/g for carbohydrates, 9 kcal/g for fats, and 4 kcal/g for proteins). Moreover, the average value of the 3-day average was calculated. The specific calculation methods are as follows:

(1) Relative value of carbohydrate energy (kcal/kg^−d−1^) = 3-day average of carbohydrate mass intake (g/^d)^ × 4 kcal/g ÷ body weight (kg). (2) Fat energy relative value (kcal/kg^−d−1^) = 3-day intake of fat mass average (g/^d^) × 9 kcal/g ÷ body weight (kg). (3) Protein energy relative value (kcal/kg^−d−1^) = 3-day intake of fat mass average (g/^d^) × 4 kcal/g ÷ body weight (kg).

### 2.4 Energy expenditure, activity intensity, and sleep efficiency

Athletes wore the ActiGraph GT3X accelerometer all day (except for showering) 1 week before the competition and 2 weeks before the competition. At the end of the period, the data from the accelerometer was imported into the accompanying software. The GT3X recorded daily energy expenditure, activity intensity, and sleep for 1 week during the training preparation and precompetition tapering periods. It also calculated the 7-day average.

### 2.5 Training real-time heart rate, respiratory rate, and heart rate variability monitoring

Athletes wore Zephyr Bioharness3.0 heart rate bands 1 week before the competition and 2 weeks before the competition. At the end of the training session, the data from the bands were exported from the accompanying software to calculate the maximum, mean, and plurality of real-time heart rate, respiratory rate, and heart rate variability for the training session.

### 2.6 Blood and urine physiological indicator tests

One week and 2 weeks before the competition, 15 mL of midmorning urine and 10 mL of fasting venous blood were collected and stored in anticoagulant tubes for subsequent testing (indicators: creatine kinase [CK], blood urea, testosterone/cortisol, hemoglobin, and urinary creatinine). Routine urinalysis was performed after the urine collection (indicators: leukocytes, glucose, occult blood, protein, nitrites, urinary bilirubin, bilirubin, ketone bodies, pH, and specific gravity).

### 2.7 Testing of mental state indicators

Athletes completed the following six questionnaires 1 week before the competition: (1) the Competition State Anxiety Inventory-2 (Martens at al., 1982), which is divided into three subscales measuring cognitive state anxiety, somatic state anxiety, and state self-confidence; (2) the Pre-Event Emotion Scale-T (Zhang, 2000), which is divided into four subscales: individual failure anxiety, self-confidence, social expectation anxiety, and somatic anxiety; (3) the Sport Competition Anxiety Scale (Martens, 1977); (4) the Cognitive Trait Anxiety Inventory for Athletics (Ping and Xiaodong, 2000); (5) Athletes’ Mental Fatigue Questionnaire First (Raedeke and Smith, 2001); and (6) Rosenberg Self-Esteem Scale (Rosenberg, 1965).

### 2.8 Statistical analysis

The results were analyzed using SPSS 26.0. The skewed distribution information was expressed as median (interquartile), and comparisons between groups were made using the nonparametric Mann-Whitney *U* test, with *P* < 0.05 being considered statistically different. All machine learning methods were executed using Python 3 and based on the scikit-learn package.

### 2.9 Data preparation for machine learning algorithms

The data were normalized and brought to the same range before being used in machine learning models. The data transformation process includes the following steps: (1) The missing values in a numerical feature are replaced by the mean of the feature range, and the missing values in a categorical feature are replaced by the plurality of values for that feature. (2) All gender-related numerical features were standardized using a Z-standardization formula based on mean and standard deviation (Equation 1): $z=\frac{x-\mu }{\sigma }$ , where µ = feature mean and σ = standard deviation. (3) Multiple rounds of random forest algorithm were implemented to reduce the feature dimensionality, thereby eliminating the features with importance less than 0.01 in each round until 20 features remain. The input data were divided into training and validation sets in the proportions of 40% and 60% for the training and validation sets, respectively, to train and validate the model.

Binary logistic regression is a classification method that generalizes logistic regression to multiclassification problems, namely, problems with more than two possible discrete outcomes. In the multinomial logistic regression model, a binary logistic regression equation was built for each category of the dependent variable. In this case, one of the categories of the dependent variable became the reference variable, and all the other categories were compared. In general, the Binary logistic regression equation can be written as Equation 2:

$$p\left(y=c|x;\theta \right)=\frac{e^{\theta_{c}^{T}x}}{\sum_{j=1}^{k}e^{\theta_{c}^{T}x}}$$

where x is the regression vector, y is the dependent variable taking the values {1, 2, . . ., k}, and q is the regression parameter determined using machine learning methods.

Random forests can be used to rank the importance of variables in a regression or classification problem in a natural way. The first step in measuring the variable importance in a data set is to fit a random forest to the data. During the fitting process, the out-of-bag error for each data point is recorded and averaged over the forest (errors on an independent test set can be substituted if bagging is not used during training). The values of the *j*th feature are permuted in the out-of-bag samples to measure the importance of the *j*th feature after training. Then, the out-of-bag error is again computed on this perturbed data set. The importance score for the *j*th feature is computed by averaging the difference in the out-of-bag error before and after the permutation over all trees. The score is normalized by the standard deviation of these differences. Features that produce large values for this score are ranked as more important than those that produce small values. The importance was calculated as follows Equation 3:

$$i\left(x\right)=\frac{1}{n_{T}}\sum_{i=1}^{n_{T}}\sum_{\text{nodej}\in T_{i}\left|\text{splitvariable}\right.\left(j\right)=x}{p_{T}}_{i}\left(j\right)\Delta {i_{T}}_{i}\left(j\right),$$

where x indicates a feature, $n_{T}$ is the number of trees in the forest, $T_{i}$ indicates tree i, ${p_{T}}_{i}\left(j\right)=\frac{n_{j}}{n}$ is the fraction of samples reaching node j, and ${{\Delta_{i}}_{T}}_{i}\left(j\right)$ is the change in impurity in tree t at node j. The proposed classification model showed resistance to noise and achieved a high accuracy of up to 100% for the training set and an accuracy of 67% for the testing set. The model could distinguish between the classes studied. The following model regularization parameters were set for training.

In machine learning, NN or ANN is a model inspired by the neuronal organization found in the biological NNs in animal brains. We selected 14 variables that are commonly important in binary logistic regression and random forest to train the NN, and the hyperparameters of the nodes and layers of the NN were optimized by setting a single hidden layer with four neurons in each layer as optimal.

## 3 Results

The two groups had no significant differences in terms of physical energy consumption, physical activity intensity, and sleep efficiency, as measured by the ActiGraph GT3X accelerometer 1 week before the competition and 2 weeks before the competition.

The two groups had no significant differences in real-time heart rate, respiratory rate, maximum, mean, and multitude of heart rate variability monitored by the Zephyr Bioharness 3.0 Heart rate bands during the preparation period for training 1 week before the competition and 2 weeks before the competition.

No significant differences were found in the relative values of the three major nutrients’ daily dietary intake for energy between the two groups 1 week before the competition and 2 weeks before the competition.

### 3.1 Variability of indicators 2 weeks before the competition

**FIGURE 1:**
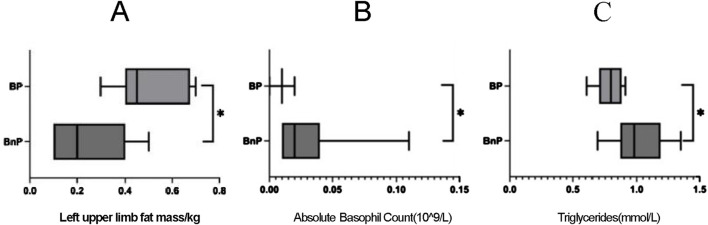
**(A)** Left upper limb fat mass differences between the two groups of players 2 weeks before the game. **(B)** Absolute Basophil Count differences between the two groups of players 2 weeks before the game. **(C)** Triglycerides differences between the two groups of players 2 weeks before the game.

During the preparation period of training, the statistical results of each index indicate that the left upper limb fat mass in the BP group was significantly higher than that in the BnP group (*P* < 0.05) in the body composition (Figure 1A). The absolute basophil count (BASO) in the BnP group was significantly higher than that in the BP group (*P* < 0.05) in the blood index (Figure 1B). Triglycerides (TGs) in the BnP group were significantly higher than those in the BP group (*P* < 0.05) (Figure 1C).

**FIGURE 2:**
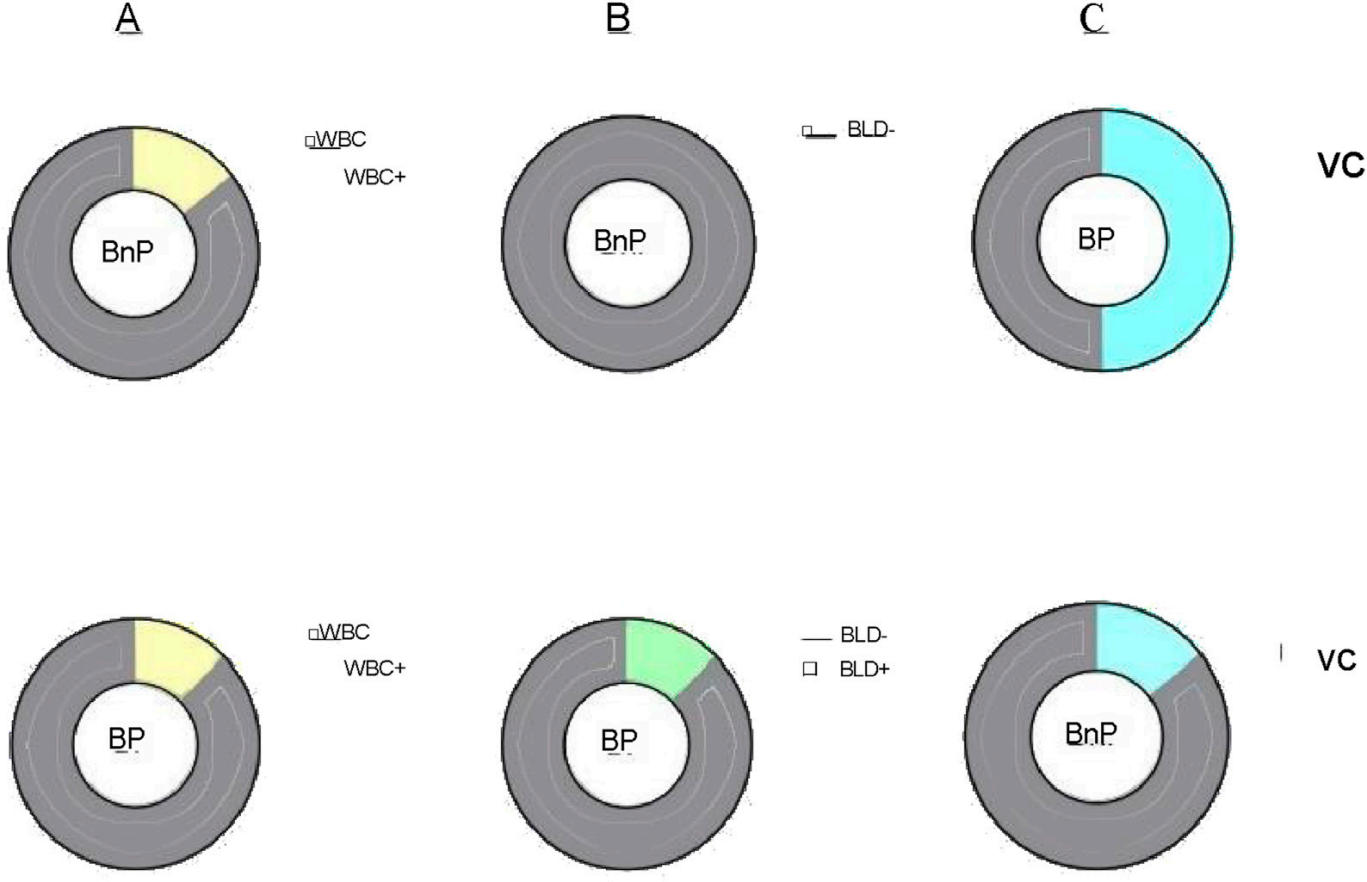
**(A)** White blood cells differences between the two groups of players 2 weeks before the game. **(B)** Urinary occult blood differences between the two groups of players 2 weeks before the game. **(C)** Vitamin C differences between the two groups of players 2 weeks before the game.

According to the statistical results of urine indexes, the positive detection rate of white blood cells (WBCs) in the BnP group was higher than that in the BP group (Figure 2A). The positive detection rate of urinary occult blood (BLD) in the BP group was higher than that in the BnP group (Figure 2B). The positive detection rate of urinary vitamin C (VC) in the BP group was higher than that in the BnP group (Figure 2C).

### 3.2 Variability of indicators 1 week before the competition

**FIGURE 3:**
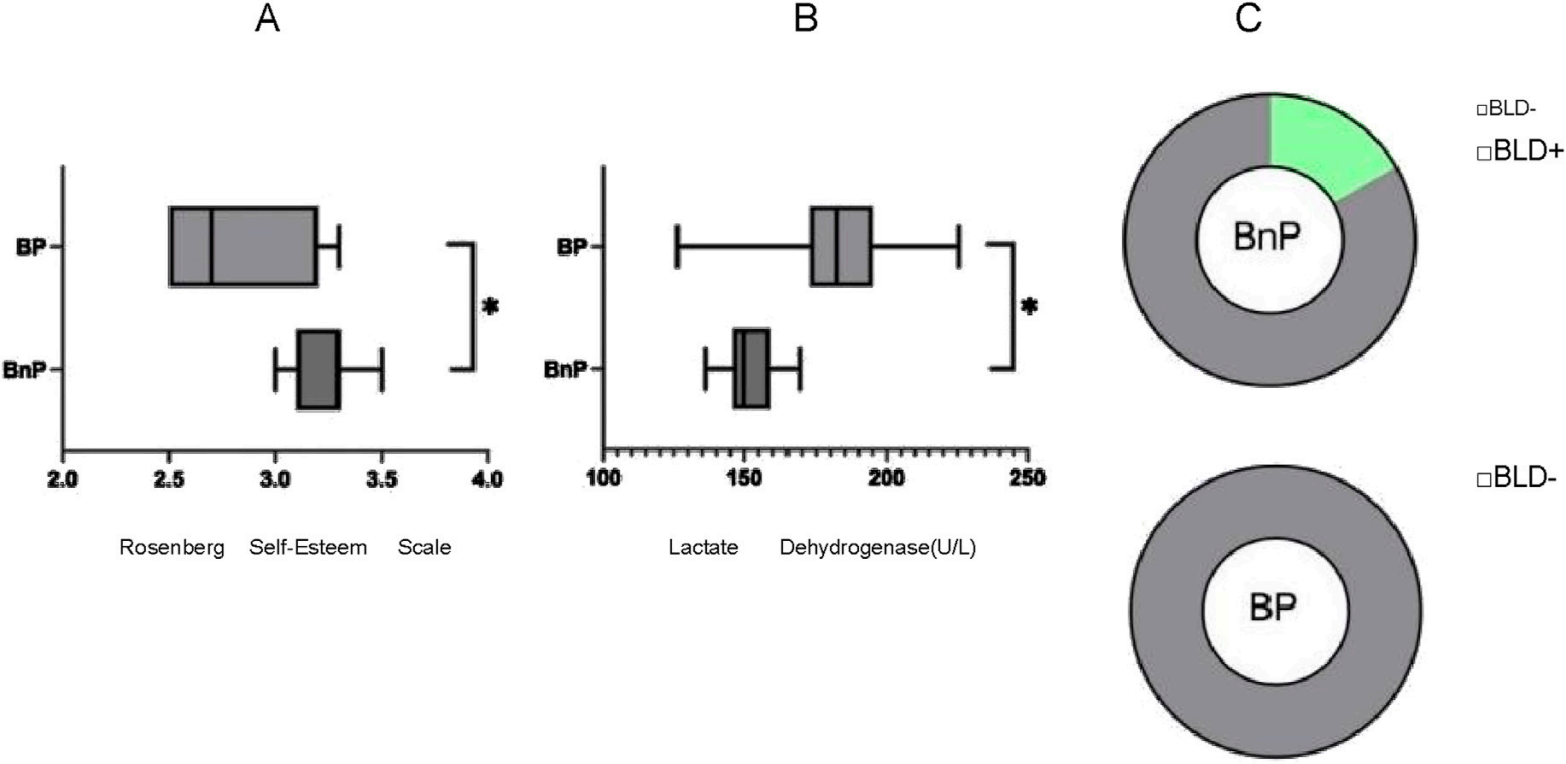
**(A)** Rosenberg Self-Esteem Scale differences between the two groups of players 1 weeks before the game. **(B)** Lactate Dehydrigenase differences between the two groups of players 1 weeks before the game. **(C)** Urinary occult blood differences between the two groups of players 1 weeks before the game.

During the precompetition reduction period, the statistical results of the psychological questionnaire showed that the results of the Rosenberg Self-esteem Scale in the BnP group were significantly higher than those in the BP group (*P* < 0.05) (Figure 3A). The results of blood indicators in the lactate dehydrogenase (LDH) in the BP group were significantly higher than those in the BnP group (*P* < 0.05) (Figure 3B). According to the statistical results of urine indicators, the positive detection rate of urinary occult blood (BLD) in the BnP group was higher than that in the BP group (Figure 3C).

### 3.3 Results of the binary logistic classification

**FIGURE 4:**
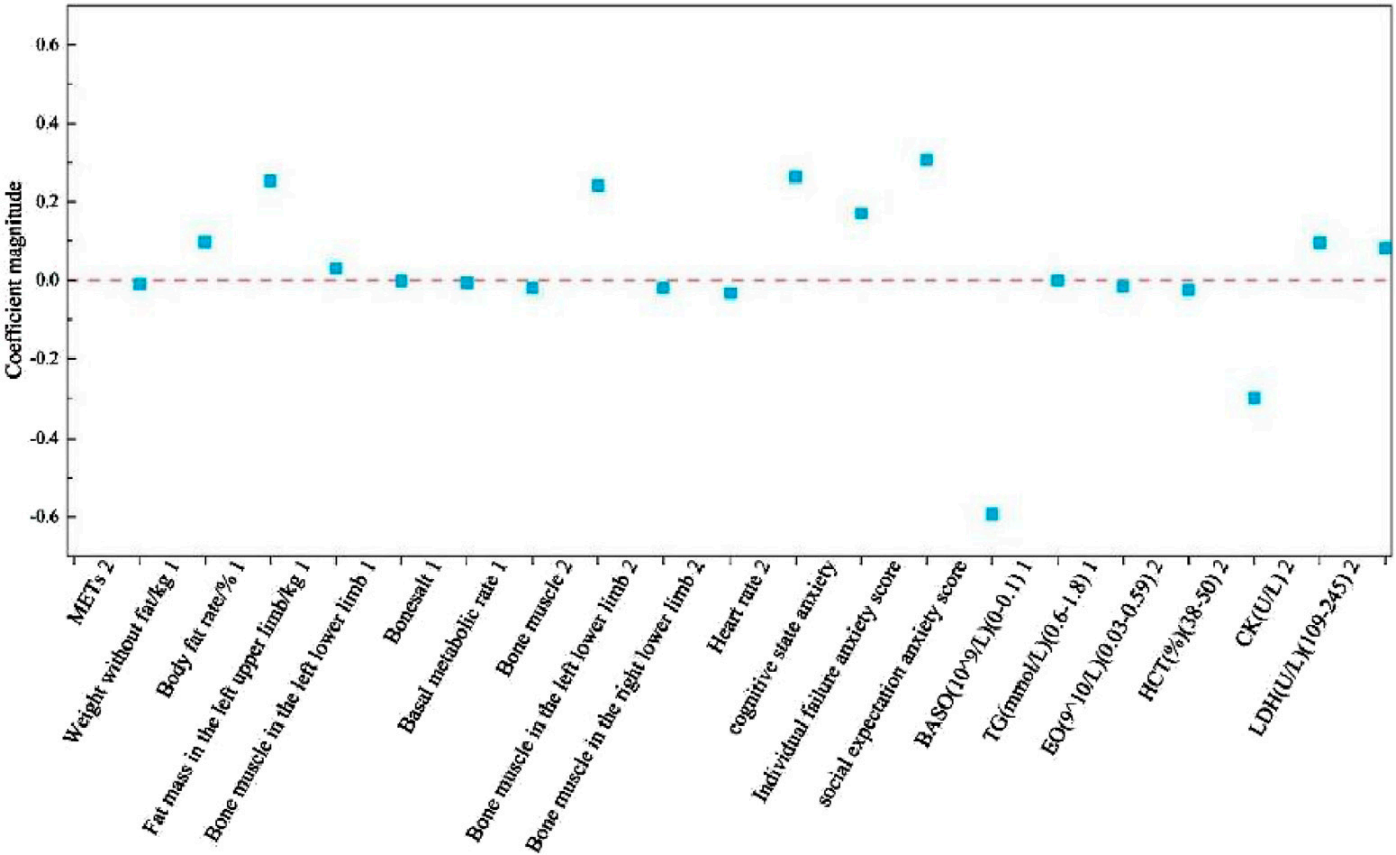
The horizontal axis shows the positive and negative correlations of each influence on athletic performance, as well as the intensity of each influence on the vertical axis; 1 stands for the intensive training period, and 2 stands for the reduction period.

The results showed that body fat rate1, bone muscle2, heart rate2, cognitive state anxiety, and Individual failure anxiety had a positive effect on the prediction of good exercise performance, whereas social expectation anxiety and hematocrit (HCT) 2 had a positive effect on the prediction of poor performance (Figure 4).

### 3.4 Random forest algorithm results

**FIGURE 5:**
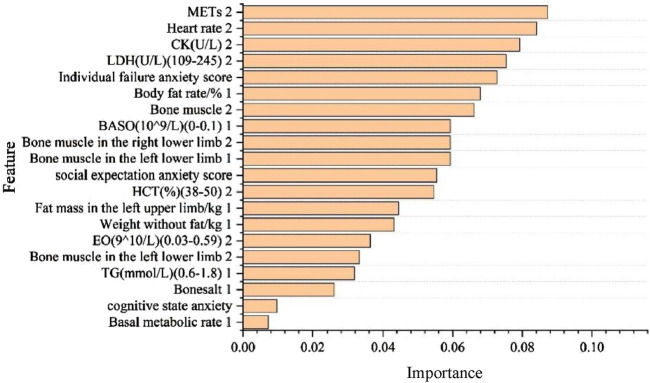
The horizontal axis shows the order of importance of each influencing factor, and the vertical axis shows each influencing factor; 1 stands for the intensive training period, and 2 stands for the reduction period.

The results showed that the order of important characteristics is as follows: METs2, heart rate2, CK2, LDH2, individual failure anxiety, body fat rate%1, bone muscle2, absolute basophil1 (BASO), bone muscle in the right lower limb2, bone muscle in the right lower limb1, social expectation anxiety, HCT2, fat mass in the left upper limb1, weight without fat1, absolute eosinophils (EOs)2, bone muscle in the right lower limb2, TG1, bonesalt1, cognitive state anxiety, and basal metabolic rate1 (Figure 5). These are the results of random forest hyperparameter optimization (Table 1).

**TABLE 1:** Random forest hyperparameter optimization results.

Number of decision trees	Maximum depth of the tree	Minimum number of samples required to split an internal node
55	4	3

### 3.5 Results of principal component analysis

**FIGURE 6:**
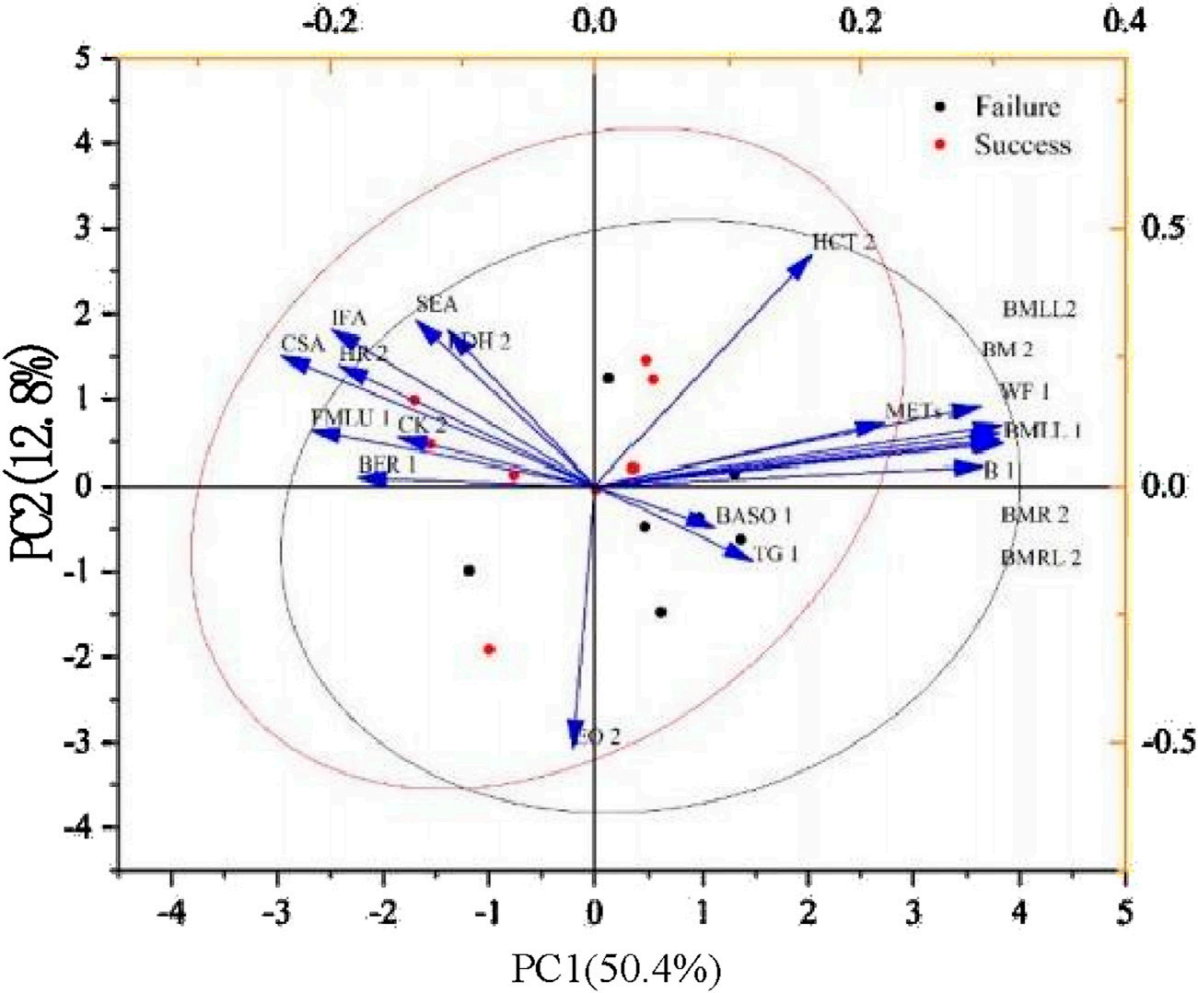
The two principal components represent the main directions of variability in the data, and the points in the graph are differentiated by two colors: black for failure (not achieving superior athletic performance) and red for success (achieving superior athletic performance). The horizontal axis is PC1, which explains 50.4% of the variability in the data. The vertical axis is PC2, explaining 12.8% of the variability. The blue arrows represent the contribution of different variables to the two principal components, and the direction and length of the arrows represent the direction and strength of the correlation of these variables with the principal components, respectively. 1 stands for the intensive training period, and 2 stands for the reduction period.

The results show that METs and body mass index (BMI) have a strong positive correlation with the difference between success (red dots) and failure (black dots). They also have a great ability to distinguish success from failure. The absolute basophil (BASO) arrow is short and points in the positive direction of the second principal component (PC2), indicating that it has a certain positive correlation with PC2. However, its explanation of the overall data variation may not be as significant as the variables on the first principal component (PC1). The two sets of indicators have differences, but many very similar features are almost equally important for classification. Moreover, interactions can be found between many features, which are slightly interpretive in explaining whether this factor affects game performance (Figure 6).

### 3.6 NN results

**FIGURE 7:**
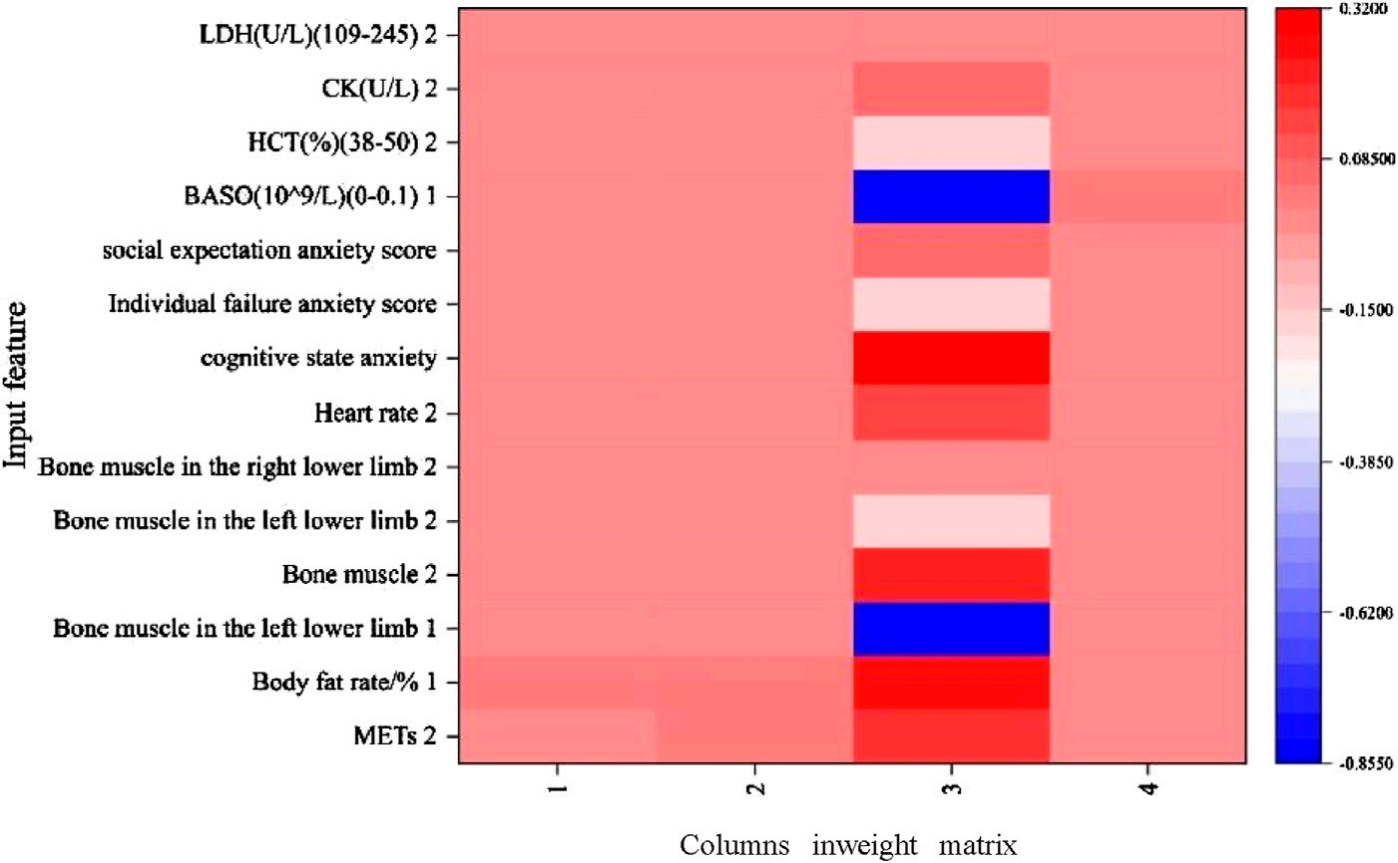
The vertical axis is for each influencing factor, and the horizontal axis represents the weights of the feature weights for a model. The color indicates the magnitude of the feature’s value in the weight matrix. The color bar shows the correspondence between the value and the color, with dark red representing positively large values (achieving superior athletic performance), dark blue representing negatively large values (not achieving superior athletic performance), and white representing values near zero. The values range from approximately −0.855 to 0.32 for the intensive training period and 2 for the reduction period.

The results show that each influencing factor has a positive impact, a negative impact, and no impact on the results of each unit. Explaining whether it has a single impact on the performance is difficult (Figure 7).

#### 3.6.1 Model comparison

**FIGURE 8:**
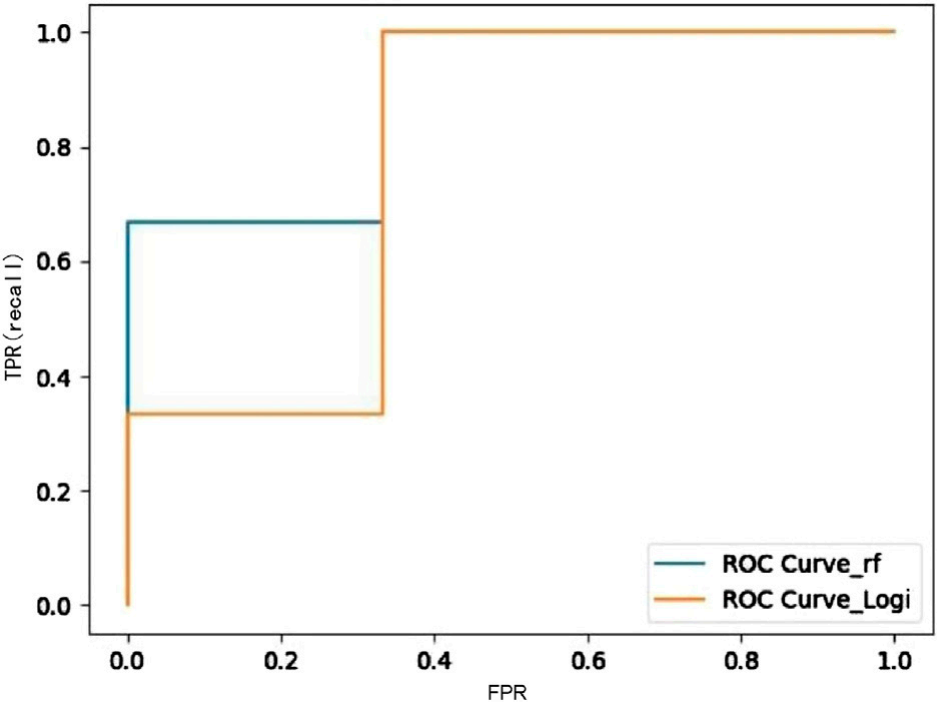
Comparison of the ROC curve for logistic and the random forest method.

The random forest method outperforms binary logistic and NNs in all indicators. The results of the receiver operating characteristic (ROC) graph also show the same conclusion (Figure 8, Table 2). The random forest method has certain advantages in feature extraction for this multifeature small sample.

**TABLE 2:** Comparison of the three machine models.

Machine learning models	Verification set accuracy	Auc	f1_score
Random Forest	0.83	0.89	0.86
Binary logistic classification	0.67	0.78	0.67
neural network	0.83	0.67	0.86

## 4 Discussion

We studied this highly efficient high-level group by comprehensively monitoring their status before the competition. We found that precompetition blood metrics, urine metrics, psychological metrics, and body composition have an impact on performance. In this study, we also used machine learning as a method to determine the key metrics affecting the performance in the competition in the hope that the result would provide guidance for student-athletes in their training and competitions. In addition, this group has long lacked specialized training monitoring. We can increase student-athletes’ and coaches’ attention to precompetition status via comprehensive precompetition monitoring (Tanner and Gore, 2012; Gronwald and Hoos, 2020; Sasmarianto et al., 2021; Lun et al., 2009; Reinebo et al., 2024; Stepanyan and Lalayan, 2023). The results of the difference analysis showed that the two groups had no significant difference in nutritional intake indicators, Zephyr Bioharness heart rate belt training real-time monitoring indicators, and GT3X accelerometer collected sleep efficiency, physical energy consumption, and activity intensity indicators. The results of the machine learning algorithm also showed that the above indicators are not important indicators affecting the performance of the race, and the consistency between the two groups of athletes in terms of training load and nutritional supplementation is high (Sermaxhaj et al., 2024). This scenario may lead to differences in the performance of the race and is related to other factors affecting the state of the precompetition.

### 4.1 Analysis of differences between the two groups of athletes 2 weeks before the competition

For athletes, alterations in body composition affect strength, endurance, speed, flexibility, and recovery, thereby affecting performance (Xu, 2021). In this study, we found that the left upper limb fat mass in the BP group was significantly higher than that in the BnP group during the training preparation period; moreover, an appropriate amount of fat is necessary to protect the joints, maintain energy reserves, and keep the body healthy in general (Che et al., 2011). A proper amount of fat reserves during the preparatory period of training is beneficial for athletes to provide energy reserves during the training period and achieve excellent training effects (Nikolaidis and Son’kin, 2023).

The impact of blood markers on exercise performance is an important area of research across multiple dimensions, including oxygenation levels, energy metabolism, nutritional status, and recovery (Wahl et al., 2020). Basophils are a type of WBC that normally plays a role in the body’s immune response. For athletes, the absolute value of EO may indirectly reflect some health conditions or physical reactions that may affect their training and performance (Lasmanova, 2014). In this study, the absolute EO values in the BnP group were significantly higher than those in the BP group; moreover, athletes may have allergies, parasitic infections, and immune disorders that affect training preparation (Chávez-Guevara et al., 2022). Lipid levels have an indirect effect on performance, but they are important indicators of cardiovascular health. Good cardiovascular health is the basis for maintaining and improving athletic performance. TGs are a form of lipid in the blood, and in this study, TGs were significantly higher in the BnP group than in the BP group. Moderate TG levels contribute to the efficient utilization and storage of energy in athletes, thereby enabling athletes to access energy reserves quickly when needed. However, high TG levels have also been associated with other hallmarks of metabolic syndrome, such as insulin resistance, hypertension, and hyperglycemia (Wang et al., 2019). Athletes in poor physical health are associated with unfavorable performance. Both groups of indicators showed differences in race performance. This finding may be related to the state of physical health during the preparatory period of training.

Urine indicators are one of the most important tools for assessing an athlete’s health and athletic performance; they can reflect hydration status, nutritional status, degree of muscle damage, and certain health problems (Bain, 2021). WBCs are part of the immune system and are responsible for fighting infections and inflammation (Damian et al., 2021), which can lead to immune dysfunction. One of its manifestations is a change in the WBC count, which affects the athlete’s performance in training and competition. WBCs are the same as basophilic leukocytes, which are one of the blood markers. The BnP group has a higher detection rate of positive leukocytes than the BP group, and the athlete has a high rate of positive leukocytes during the training preparation period. Problems in health status during the preparatory period of training have an impact on the training effect of the subsequent training (Pan and Wang, 2020), thereby affecting the performance of the game. In athletes, sports training load may lead to the emergence of abnormal excretions, such as proteinuria and urinary occult blood. In this study, the positive detection rate of urinary occult blood (BLD) in the BP group is higher than that in the BnP group. This result may be due to the fact that the BP group had a high training load in the previous phase, and the athletes had not fully recovered (Sabzevari Rad, 2023). Urinary VC varies significantly among athletes with different performance levels with high impact weights. A placebo study has shown that 500 mg of VC before and after exercise reduces the risk of upper respiratory infections (Tiller et al., 2019). Studies have shown that the risk of upper respiratory infections is lower in athletes with different performance levels. Plasma VC concentrations were significantly lower 2–4 days after completion of a half-marathon. The body consumes VC to reduce oxidized LDL cholesterol and oxidized vitamin E (Farhana and Lappin, 2020). The results of the present study showed that the positive detection rate of urinary VC in the BP group was higher than that in the BnP group, suggesting that the athletes in the BP group had a better physical condition than those in the BnP group.

### 4.2 Analysis of differences between the two groups of athletes 2 weeks before the competition

The previous sections discuss that blood indicators are important in examining the physical status of athletes. LDH is an enzyme widely found in the body and plays a key role in the metabolism of lactic acid. During a high-intensity exercise, the supply of oxygen to the muscle cells may not be sufficient to meet the energy demand, leading to an increase in anaerobic metabolism and the accumulation of lactate and H+ ions. LDH converts lactate to pyruvate in this process, thereby helping to maintain intracellular acid–base balance and delaying the onset of muscle fatigue. Therefore, the activity level of LDH can reflect the anaerobic endurance capacity of athletes to a certain extent (Dao, 2021). After exercise, the removal of lactic acid from the body is an important part of the recovery process. LDH plays a key role in the conversion of lactate back to pyruvate. This process not only helps to eliminate lactate quickly but also promotes the regenerative utilization of energy. The effective activity of LDH is essential for rapid recovery after exercise. The results of this study showed that the LDH of the BP group was significantly higher than that of the BnP group. The enhanced lactate metabolism and recovery ability and the improved adaptability during training are conducive to producing excellent athletic performance (Gibb et al., 2022; Borresen and Lambert, 2009).

The statistical results of urine indexes show the effects of urinary occult blood (UOB) on athletes’ physical functions. According to the results of the study, the positive detection rate of UOB in the BnP group during the precompetition reduction period was higher than that in the BP group. The exercise load affects UOB, and the athletes in the BnP group have a high exercise load before the competition, thereby affecting their exercise performance (Lopes Dos Santos et al., 2020).

### 4.3 Analysis of differences in psychological questionnaire indicators

The results of the Rosenberg Self-esteem Scale reflect an individual’s level of self-esteem, which affects an athlete’s athletic performance in several ways. Self-esteem refers to an individual’s overall assessment of self-worth, including feelings of self-acceptance and self-respect. Athletic performance is influenced not only by physical training and skills but also by psychological factors, such as the athlete’s mental state, emotional regulation, and self-perception. Athletes with high self-esteem typically possess great self-confidence and self-efficacy in their ability to reach goals and overcome challenges. This belief helps them to stay focused and calm in the face of competition and stress; as a result, their athletic performance can be improved. The results of the Rosenberg Self-Esteem Scale in the BnP group in this study were significantly higher than those in the BP group. Some studies have shown that high self-esteem may sometimes translate into overconfidence or complacency. Athletes who maintain an overconfident and complacent mentality do not work hard enough in their preparation and training, and this attitude affects their athletic performance. Athletes may also face expectations and pressures from coaches, teammates, and family. Even if they have high self-esteem, the pressure of these external factors may have a negative impact on their performance in important competitions (Powell et al., 2022). Coaches should provide individualized training and psychological support to each athlete according to his or her specific needs to help this individual build on his or her high self-esteem and enhance other psychological and physical factors that have a significant impact on athletic performance (Couronné et al., 2018).

### 4.4 Analysis of machine learning results

In analyzing the weights of the influencing factors affecting race performance, we used multinomial logistic regression with a random forest algorithm in machine learning analysis methods. The multinomial logistic regression screened for significant overlap with the traditional rank-sum test of variance for absolute basophil (BASO) during the training preparation period and LDH during the prerace tapering period. The random forest algorithm screened for significant overlap with the traditional rank sum test for fat mass in the left upper limb, absolute basophil (BASO), TGs, LDH, and LDH during the precompetition taper period. For LDH, the high rate of overlap suggests a high degree of agreement between the traditional rank-sum test and the machine learning algorithm in determining the influences on race performance and increasing confidence in the influences in the study. In addition. multinomial logistic regression, which has a high overlap with the random forest algorithm for determining the importance of influencing factors, presents body fat rate and absolute basophil (BASO) during the preparation period. CK, LDH, erythrocyte pressure (HCT), heart rate, and other factors can be used to judge the importance of the factors in the precompetition tapering period. For the CK, LDH, HCT, heart rate, and individual failure anxiety, a high degree of agreement exists between logistic regression and random forests in identifying influences. Logistic regression and random forests have a high degree of agreement in identifying influencing factors. However, they differ in their methodology, the way they handle the data, and their ability to interpret the model. Logistic regression provides a direct explanation of the effects of the influencing factors on the probability of an outcome, whereas random forests can deal with nonlinear relationships and interactions between features through their integrated learning properties (Elhaik, 2022; Isomura and Toyoizumi, 2021). The high rate of overlap suggests that despite their different methodological approaches, they can complement one another in dealing with issues related to influencing factors affecting performance. This high degree of congruence provides a solid basis for the present study of athletes’ precompetition status that can affect their performance.

In principal component analysis plots, two groups of different but very similar features are of almost equal importance for classification, and interactions exist between many of the features. Given the interactions, determining from the results that any feature is the most important is difficult (Achterberg et al., 2023). We should consider using PCA to reduce dimensionality and create new features to obtain excellent predictive models in the future [40]. The overall PCA helps with minimal explanatory predictions, and the algorithm for addressing the weighting of the influencing factors in this study is barely appropriate.

An NN refers to the relationship between the input features and the weight matrix of the influencing factors in predicting an athlete’s performance in a competition. Each cell color in the heat map represents the size of a particular input feature corresponding to a certain weight in the NN, which affects the output of the model. The results of this study show that a feature has a small weight on each cell, and that feature is the least important for the model. As a result, explaining which influencing factor is the least important becomes difficult (Achterberg et al., 2023). NNs are hardly suitable for the study of weights of the influencing factors in small sample studies. However, they are informative in predicting the performance of the competition through influencing factors, which is difficult to interpret.

## 5 Conclusion

The precompetition assessment of absolute basophil count (BASO), LDH levels, TG concentrations, WBC count, CK activity, fat mass in the left upper limb, HCT, and individual failure anxiety can serve as a comprehensive training monitoring indicator. This indicator set is particularly focused on tracking athlete status before competitions to provide valuable insights into their physiological and psychological preparedness.

## Data Availability

The raw data supporting the conclusions of this article will be made available by the authors, without undue reservation.
